# From symptom onset to diagnosis: a critical exploration into the experiences of young people with juvenile systemic lupus erythematosus (JSLE)

**DOI:** 10.1186/1546-0096-12-S1-P317

**Published:** 2014-09-17

**Authors:** Olivia Lloyd, Lyvonne Tume, Michael Beresford, Bernie Carter

**Affiliations:** 1 Children's Nursing Research Unit, NHS, Liverpool, UK; 2 Department of Paediatric Rheumatology, NHS, Liverpool, UK; 3 School of Health, University of Central Lancashire, Preston, UK; 4 Institute of Translational Medicine (Child Health), University of Liverpool, Liverpool, UK

## Introduction

There is a paucity of literature relating to the experiences of young people before and during diagnosis of JSLE. An understanding of young people’s experiences and the issues relevant to them could help improve outcomes and facilitate the development of standards of care in JSLE.

## Objectives

- To describe the journey from onset of symptoms to diagnosis in JSLE from a young person’s perspective by exploring the stories they tell.

- Ascertain key points if any in the journey to diagnosis to generate deeper insight into access to care for young people with SLE.

## Methods

An exploratory qualitative approach was used in one UK centre for paediatric rheumatology with young people who were participating in the UK JSLE Cohort Study and who had received their diagnosis after January 2010. The study utilised in-depth interviews with eight young people who told their story of their own ‘journey to diagnosis’. Data were subjected to thematic analysis (Braun & Clarke 2006). All ethical issues were addressed.

## Results

Four themes were generated and are linked by a meta-theme ‘passing of time’. This was not a static concept; it moved slowly in the first two themes but was experienced as a single point in time in theme 3.

1. ‘Emerging Illness’ encompasses the first descriptions of a change in health, the emergence of physical symptoms and the impact of these symptoms on the young people’s lives. The main physical symptoms were tiredness, pain, rash, sickness and other non-specific symptoms. These symptoms gradually affect their lives preventing them from doing ordinary things. Often symptoms were dismissed or ignored.

2. ‘Seeking Help’ lasted from weeks to up to 2 years and covered the first and ongoing contacts with health services. All first contacts were with a General Practitioner; most young people experienced dissatisfaction with how their symptoms were dealt with.

3. ‘Diagnosis of Lupus’ was a significant time point as the young people experienced a change in health status. For some, diagnosis was a relief; others were worried by the implications of the condition. They all talked about how their diagnosis impacted on them and how it affected their family.

4. ‘Resilience, Reflection & Recovery’ encompasses the experiences that had occurred after diagnosis. It is characterised by things that went well and things that did not.

## Conclusion

This study reveals how young people may not recognise symptom-related changes. It also shows the struggle they/their parents often have in being taken seriously when they seek help. All young people should have the opportunity to tell their ‘journey to diagnosis’ story to a care team member. This would allow experiences to be contextualised and support offered. Raising awareness about emerging symptoms of lupus and increasing support after diagnosis is also recommended. Understanding young people’s experiences of 'symptom onset to diagnosis' has the potential to reduce the impact and burden of this disease.

## Disclosure of interest

None declared

